# Laser therapy as a care technology in primary health care

**DOI:** 10.1590/1980-220X-REEUSP-2024-0249en

**Published:** 2025-03-24

**Authors:** Graziela de Paula Povrezan, Luzana Mackevicius Bernardes, Eduardo Carvalho de Souza, Rosimeire Angela de Queiroz Soares, Elda de Oliveira

**Affiliations:** 1 Universidade Católica de Santos, Programa de Pós-Graduação Stricto Sensu em Saúde Coletiva, Santos, SP, Brazil.; 2 Faculdade de Ciências Médicas da Santa Casa de São Paulo, São Paulo, SP, Brazil.

**Keywords:** Laser Therapy, Primary Health Care, Nursing Care, Organization and Administration

## Abstract

**Objective:**

To report on the experience of implementing low-power laser therapy in Primary Health Care (PHC) in the municipality of Santos.

**Method:**

Descriptive study with a time frame from 2017 to 2024. The participants were nurses (specialists and general practitioners) and managers. The analysis was based on the theoretical framework of clinical management.

**Results:**

The implementation of laser therapy began with one nurse in a small hospital. The success of the process was driven by the nurse’s political articulation, dialog with different levels of care and managers, and the acquisition of laser equipment. A total of 115 nurses were qualified to perform the technique in all SUS units in the municipality, with an emphasis on PHC.

**Final considerations:**

The experience demonstrates the potential of local initiatives to transform clinical management in the SUS. Collaboration between managers, health professionals, and the community is crucial for a more efficient, accessible, and humane health system.

## INTRODUCTION

The treatment of chronic wounds is a global public health challenge, impacting millions of individuals and generating significant costs for health systems. It is estimated that the prevalence of chronic wounds ranges from 0.18% to 1.3% of the population, depending on the context and diagnostic criteria used^(1)^. These lesions are often associated with chronic diseases such as diabetes and vascular diseases, and impose considerable discomfort on the subjects, compromising their quality of life and increasing the risk of serious complications such as infections and amputations. It is, therefore, a priority in healthcare to establish effective therapies that promote healing and minimize suffering. Thus, Low Power Laser Therapy (LPLT) has emerged as a promising technological innovation in this scenario, with the potential to significantly contribute to the clinical management of wounds^(2)^.

Research in the use and development of technologies and innovation in health has been encouraged since 2000, with the creation of the Department of Science and Technology, part of the Secretariat of Science, Technology, and Strategic Inputs. In 2018, this department, and the Pan American Health Organization launched the Ministry of Health’s new research priority agenda^(3)^. Axis four of the agenda deals with the importance of conducting research in the field of technology development and innovation in health, especially in Primary Health Care^(3)^, the preferred gateway and organizer of care in the Unified Health System, with primary functions for integrating the Health Care Network (HCN)^(4)^. So LPLT, one of the technological innovations, has been implemented in the care of Primary Health Care users^(5,6)^.

LPLT has been used worldwide since the 1980s, when Tina Karu, an important Estonian biophysicist, demonstrated low-power light’s effects on molecular components. When they absorb the light beams, these molecular components show short-term activation of electron transport, increasing the synthesis of adenosine triphosphate and reducing intracellular pH. These physiological reactions form the conceptual basis of LPLT’s effects, leading the cell to a condition of equilibrium^(7)^.

The action of LPLT favors the healing process and has a bactericidal action. The advantages of the clinic include pain relief, control of inflammatory processes, treatment of infections, tissue regeneration, and edema drainage, with the intervention varying according to the person’s care needs. In addition, the use of LPLT reduces hospital costs in the treatment of skin lesions^(8)^.

In 2017, the use of LPLT began in the municipality of Santos, with a nurse (stomatherapist and hospital manager) who started using this therapy with her equipment to treat skin lesions in a hospital complex in the municipality. The successful experiences of this nurse highlighted the need to expand this intervention in Primary Health Care. The implementation of LPLT in care within the Health Care Network aligns with its proposal to offer care services that involve promotion, prevention, diagnosis, treatment, and rehabilitation in collective and individual health. Therefore, this text aims to report on the experience of implementing LPLT in primary health care in the municipality of Santos.

## METHOD

The following is a descriptive study of the experience report type. When socializing an experience, a critical-reflective approach is needed to spark debates and enable reflections, contributing to the production of scientific knowledge^(9)^. The description is temporal and cross-sectional, covering 2017 to 2024.

The theoretical framework for the analysis is based on clinical management^(10)^, which highlights three structuring axes in integrated health systems, such as the health care network: the healthcare model, the management model, and the conception of health education. These axes provide a solid basis for implementing effective and coherent health care practices and guide the principles of clinic management in a democratic and co-participatory administration.

The experience reported began in a small hospital complex in the municipality of Santos. Subsequently, the initiative was widely disseminated throughout the municipality, located on the coast of the state of São Paulo, 70 km from the capital. Santos has an estimated population of 433,656 inhabitants and a human development index of 0.840; 32 primary care units and nurses work in all of them. The city is divided by the IBGE into eight health district areas. Its inhabitants, when compared to the rest of the state of São Paulo, are an older population, i.e., over 60 years old^(11)^, corresponding to 22.6% of the population.

As mentioned above, a nurse coordinated the bureaucratic process of implementing laser therapy, which included fundamental steps to guarantee its execution and quality. The initiative had the support of the municipal health administration and a budget obtained through parliamentary amendments intended to fund the project. This careful planning was essential to ensure that the program content was structured to standards of excellence.

As this is an experience report, no information would allow people or institutions to be identified, and there is no need to submit it to the Ethics Committee.

## RESULTS

The implementation of LPLT in Santos was driven by the promising results observed in its initial application in a small hospital complex. Based on this experience, a situational diagnosis was carried out to identify the number of people needing laser therapy and the number of professionals who could be trained. Based on this survey, a bidding process was carried out in the municipality of Santos to hire an education service for the qualification and training of nurses in the use of laser equipment, by the opinion of the Technical Chamber Number 04/2016/Ctas/Cofen^(12)^ and the opinion of the Nursing Council of Sao Paulo (COREN-SP) Number 009/2018^(13)^ on the use of red and infrared lasers in the treatment of wounds carried out by nurses.

The Municipal Health Department managed the aforementioned parliamentary amendment, and the training was held at a university. The course lasted 30 theoretical and practical hours and covered the prophylactic and curative treatment of acute and chronic tissue lesions with LPLT.

The content developed and taught in this training covered the following topics: indications and contraindications for laser use; understanding light; energy density or fluence; light and interaction with biological tissue; photodynamic laser therapy; laser therapy in the treatment of pain; laser therapy in bone growth; biosafety standards; ILIB (Intravascular Laser Irradiation of Blood); healing and pain protocols; laser therapy *versus* coverings; equipment available in Brazil; laser therapy in children; laser therapy in postpartum injuries; oral injuries and laser therapy; aesthetics; surgical laser; and strategies for implementing laser therapy and defining values associated with the practice. This approach ensured that the participants had access to up-to-date knowledge and high- quality tools for application in the professional field.

Thirty-three nurses were selected to participate in the training, prioritizing those working in units with the highest demand for dressings. After the training, these professionals were assigned to nine health units, representing an initial coverage of 28% of the municipal territory. At the same time, a dressing center was set up at the dermatology and stomatherapy outpatient clinic in the municipality of Santos to deal with more complex cases.

The expansion of LPLT to all primary health care units was carried out gradually, driven by the positive results observed and the coordination of the nursing team with municipal managers. In 2019, two years after the nurses’ first training process, the need to expand LPLT to all primary care units in the municipality of Santos became evident. The successful experiences of the initial project were presented to managers, such as the reduction and shortening of hospitalization times when the laser was used to heal wounds, as well as providing more agile and capillary care, responding more effectively to the high demand from users with acute or chronic tissue injuries.

In the process, Bill No. 233/2019^(14)^ was drafted, limited to care for people with vascular diseases and injuries resulting from these pathologies throughout the municipality of Santos. As a result, the Santos Health Department offered funds to expand the project, enabling the purchase of two more pieces of equipment, bringing the total to 11 health units with laser equipment, covering 34% of the municipality’s land area. That same year, the first Dressings Symposium was held in the city.

Based on the success of the initial stage, a new tender was held in 2023. Two new training courses were held, enabling another 82 nurses to participate. The training was divided into two groups: 41 in the first and 41 in the second. In total, 115 nurses (33 in the first training and 82 in the second training) who work in the health care network in the municipality of Santos were qualified to manage LPLT, strengthening the care network and expanding the impact of the training activities.

The result was the inclusion of trained nurses in all of the municipality’s primary care units, making it possible to use LPLT to care for people with hard-to-heal wounds in specially dedicated rooms. With this in mind, currently, 100% of the territory, including the two hospitals and the three specialty outpatient units, is covered by LPLT care.

As a result of these actions, Municipal Law No. 4,483 of May 14, 2024^(15)^ was created, establishing the municipal vascular care program. The aim is to promote the expansion of public policies aimed at the prevention and treatment of vascular diseases, providing qualified, specialized, and humanized care to people affected by vascular diseases and resulting injuries through the municipality’s dressing center, which now has a vascular surgeon and specialized nurses from the municipal network.

It is worth noting that all the municipal institutions in which LPLT is offered are interconnected. The nurses receive ongoing training through continuing education to standardize and standardize techniques so that care is carried out according to what is learned in the training offered. In the training sessions, the approaches focused on the most common types of wounds in the services, which varied according to the specialties: in vascular services, ulcers, and diabetic foot wounds predominate, while in pediatrics and maternal and child health, the boils were more frequent.

A protocol is currently under construction and will be printed as an official document by the Health Department to guide professional practices and conduct in the municipality.

## DISCUSSION

The municipality of Santos is a pioneer in the state of São Paulo and Brazil in implementing laser therapy technology in 100% of primary health care units. This experience reorients health care according to the needs of individuals (users/patients) or social groups. Communication with the entire healthcare network, including its administrators, is necessary^(10)^. As the case presented shows, that is only possible due to the relative autonomy granted to municipal administrators by the decentralization of power offered by the SUS policy.

In this model of democratic and co-participatory management, municipal managers act as agents in implementing policies and incorporating new technologies into health practices. These organizational planning and management measures involve organizing the municipal service network and responding to the individual and collective health needs of the people in their municipality^(10,16)^. This autonomy is essential for power distribuition and co-responsibility between managers, health professionals, and citizens in providing care. In the Santos municipality’s experience, the decision-making process was shared between workers and managers, guiding care according to health needs and comprehensive care^(10)^.

In this context, there was linkage and communication between professionals at the different levels of care, with primary care as the main point of care in the network. Primary care aims to be resolutive and free of prejudice (racial, sexual, religious, of origin), serving the territory’s population and playing a fundamental role in clinical management^(17)^. In this direction, it is considered to integrate and legitimize the various health knowledge and practices to face the challenges and with the proposal of health education for professionals in their work processes, through permanent education, also considering the reality where they are inserted^(18)^. In this way, the objective of the health care network is fulfilled: comprehensive and humanized health care within the SUS^(17)^.

Improving healthcare is also driven by new technologies, which promote efficiency, accessibility, and quality of services in various lines of care. In this sense, LPLT represents the latest technology implemented in the municipality of Santos, aimed at the care of chronic non-communicable diseases, especially for people with vascular diseases and injuries resulting from diabetes^(15)^, for example. Many chronic non-communicable diseases cannot be cured, and the same can happen with wounds that cannot be cured, in which case people with these wounds are also called “chronic patients”; however, regardless of the potential for cure, these people can and should receive appropriate and timely treatment in order to maintain their autonomy^(1)^.

This experience made it possible to expand the portfolio of primary healthcare services in the municipality of Santos. It is a document that guides health actions in Brazilian primary care, with strong recognition of the clinic. Therefore, the experience presents LPLT as an innovative technology for health care that has already been included in the SUS^(19)^. In addition, this therapy strengthens primary care’s ability to resolve problems.

This technological innovation and the actions of the nurses and the team prevent people from circulating between services in search of care, creating a fragmentation of health care. In this process, it is important to emphasize that the nurse and the team offer shared and singular care for the subject. If the patient needs more complex care, the care plan (treatment) is shared between the person being cared for, their family, the community, and managers; this is done through accessible and timely dialog to improve care^(10,20)^.

Therefore, this report does not contain numerical data on care, as it aims is to discuss the process of implementing the new technology in the SUS. The role played by nurses in implementing LPLT demonstrates that technological innovation, combined with professional commitment and participatory management, can transform the reality of primary health care, promoting comprehensive, problem-solving care centered on the population’s needs.

## CONCLUSION

The relevance of this report is the initiative led by the nurses, which not only exemplifies the autonomy and capacity for innovation of professionals but also reflects the importance of political and organizational involvement in improving health services. The nurses’ leadership was crucial to the introduction of LPLT, demonstrating that decentralizing power in the SUS allows implementing solutions tailored to health needs according to the municipality. This proactive approach expanded the portfolio of Primary Health Care services and strengthened integration between the different levels of care, promoting comprehensive and resolutive care.

Using LPLT as a new technology for hard-to-heal wounds demonstrates how technological innovations can be effectively incorporated into daily clinical practice, improving people’s quality of life and optimizing health system resources. The ongoing education of nurses ensures continuity and quality of care, ensuring the new technology is used effectively and safely.

The Santos experience could serve as a reference model for other regions and illustrate the potential of local initiatives to transform clinical management in the SUS. Collaboration between managers, health professionals, and the community is essential for creating a more efficient, accessible, and humane health system.
