# Supplementary material for: Laser therapy as a care technology in primary health care
Source: Rev Esc Enferm USP. 2025 Mar 24;59:e20240249. doi: 10.1590/1980-220X-REEUSP-2024-0249en (PMC11932700; doi:10.1590/1980-220X-REEUSP-2024-0249en)
Supplement: Translation

# Translation: Laserterapia como tecnologia do cuidado na atenção primária à saúde

**DOI:** 10.1590/1980-220X-REEUSP-2024-0249pt

**Authors:** Graziela de Paula Povrezan, Luzana Mackevicius Bernardes, Eduardo Carvalho de Souza, Rosimeire Angela de Queiroz Soares, Elda de Oliveira

**Affiliations:** 1 Universidade Católica de Santos, Programa de Pós-Graduação Stricto Sensu em Saúde Coletiva, Santos, SP, Brasil.; 2 Faculdade de Ciências Médicas da Santa Casa de São Paulo, São Paulo, SP, Brasil.

**Keywords:** Terapia a Laser, Atenção Primária à Saúde, Cuidados de Enfermagem, Organização e Administração

## Abstract

**Objetivo:**

Relatar a experiência da implementação da terapia a laser de baixa potência na Atenção Primária à Saúde (APS) no município de Santos.

**Método:**

Estudo descritivo com recorte temporal de 2017 a 2024. Participaram enfermeiras (especialistas e generalistas) e gestores. A análise foi baseada no referencial teórico da gestão da clínica.

**Resultados:**

A implementação da laserterapia teve início com uma enfermeira em uma instituição hospitalar de pequeno porte. O êxito do processo foi impulsionado pela articulação política da enfermeira, pelo diálogo com diferentes níveis de atenção e gestores e pela aquisição dos equipamentos de laser. Foram habilitadas 115 enfermeiras para execução da técnica em todas as unidades do SUS no município, destacando a APS.

**Considerações finais:**

A experiência demonstra o potencial das iniciativas locais para transformar a gestão da clínica no SUS. A colaboração entre gestores, profissionais de saúde e comunidade é crucial para um sistema de saúde mais eficiente, acessível e humano.

## INTRODUÇÃO

O tratamento de feridas crônicas representa um desafio global de saúde pública, impactando milhões de indivíduos e gerando custos significativos para os sistemas de saúde. Estima-se que a prevalência de feridas crônicas varie entre 0,18% e 1,3% da população, dependendo do contexto e dos critérios diagnósticos utilizados^(1)^. Essas lesões, muitas vezes, são associadas a doenças crônicas como diabetes e doenças vasculares, e impõem um desconforto considerável aos sujeitos, comprometendo sua qualidade de vida e aumentando o risco de complicações graves, como infecções e amputações. Portanto, é uma prioridade na área da saúde estabelecer terapias eficazes que promovam a cicatrização e minimizem o sofrimento dos sujeitos. Assim, a Terapia a Laser de Baixa Potência (TLBP) surge como uma alternativa tecnológica de inovação promissora nesse cenário, com o potencial de contribuição significativa no gerenciamento clínico de feridas^(2)^.

Pesquisas no campo do uso e desenvolvimento de tecnologias e inovação em saúde vêm sendo incentivadas desde 2000, com a criação do Departamento de Ciência e Tecnologia, integrante da Secretaria de Ciência, Tecnologia e Insumos Estratégicos. Em 2018, esse departamento, juntamente com a Organização Panamericana da Saúde, lançou a nova agenda de prioridade de pesquisa do Ministério da Saúde^(3)^. O eixo quatro da agenda trata da importância da realização de pesquisas no campo do desenvolvimento de tecnologias e inovação em saúde, sobretudo na Atenção Primária à Saúde^(3)^, porta preferencial de entrada e ordenadora do cuidado no Sistema Único de Saúde, com funções primordiais para integração da Rede de Atenção à Saúde (RAS)^(4)^. De modo que a TLBP, uma das inovações tecnológicas, vêm sendo implementada no cuidado aos usuários da Atenção Primária à Saúde^(5,6)^.

Globalmente, a TLBP vem sendo utilizada desde a década de 1980, quando Tina Karu, uma importante biofísica de origem estoniana, demonstrou os efeitos da luz de baixa potência em componentes moleculares. Esses componentes moleculares, quando absorvem os feixes de luz, apresentam ativação de curto prazo do transporte de elétrons, aumentando a síntese do trifosfato de adenosina e reduzindo o pH intracelular. Essas reações fisiológicas formam a base conceitual dos efeitos da TLBP, levando a célula a uma condição de equilíbrio^(7)^.

A ação da TLBP favorece o processo de cicatrização e a ação bactericida. As vantagens na clínica incluem o alívio da dor, controle de processos inflamatórios, tratamento de infecções, regeneração de tecidos e drenagem de edemas, com a intervenção variando de acordo com as necessidades de cuidado da pessoa. Além disso, o uso da TLBP diminui os custos hospitalares nos tratamentos de lesões cutâneas^(8)^.

Em 2017, o início o uso da TLBP no município de Santos foi implementado por uma enfermeira (estomaterapeuta e gestora hospitalar) que passou a utilizar essa terapia, com equipamento próprio, para tratar lesões cutâneas em um complexo hospitalar do município. As experiências exitosas desta enfermeira, evidenciaram a necessidade de expansão dessa intervenção na Atenção Primária à Saúde. A implementação da TLBP no cuidado na Rede de Atenção à Saúde está alinhada com sua proposta de oferecer serviços de atendimento que envolvam promoção, prevenção, diagnóstico, tratamento e reabilitação em saúde coletiva e individual. O objetivo deste texto é, portanto, relatar a experiência da implementação da TLBP na Atenção Primária à Saúde no município de Santos.

## MÉTODO

Trata-se de um estudo descritivo do tipo relato de experiência. Ao socializar a experiência, faz-se necessária uma abordagem crítico-reflexiva que suscite o debate e viabilize reflexões, contribuindo para a produção do conhecimento científico^(9)^. O recorte da descrição é temporal e transversal, compreendendo o período 2017 a 2024.

O referencial teórico para a análise, baseia-se na gestão da clínica^(10)^, que destaca três eixos estruturantes em sistemas integrados de saúde, como a rede de atenção à saúde: o modelo de atenção à saúde; o modelo de gestão e a concepção de educação na saúde. Esses eixos fornecem uma base sólida para a implementação de práticas eficazes e coerentes no atendimento à saúde e orientam os princípios da gestão da clínica em uma administração democrática e coparticipativa.

A experiência relatada teve início em um complexo hospitalar de pequeno porte do município de Santos. Subsequentemente, uma ampla disseminação da iniciativa contemplou todo o município, que está localizado no litoral do Estado de São Paulo, a 70 km da capital. Santos possui população estimada em 433.656 habitantes e o índice de desenvolvimento humano é de 0,840; possui 32 unidades de atenção primária e em todas as unidades há enfermeiras atuando. A cidade é dividida, pelo IBGE, em oito áreas ou distritos de saúde. Os habitantes, quando comparados aos demais Estado de São Paulo, constituem-se como uma população mais envelhecida, isto é, com mais de 60 anos^(11)^, correspondendo a 22,6% da população.

O processo de implementação de laserterapia, como supracitado, foi coordenado por uma enfermeira que viabilizou o trâmite burocrático, e incluiu etapas fundamentais para garantir sua execução e qualidade. A iniciativa, contou com o apoio da gestão municipal de saúde e com orçamento obtido por aprovação de emendas parlamentares, destinadas ao financiamento do projeto. Esse planejamento criterioso, foi essencial para assegurar a estruturação do conteúdo programático com padrões de excelência.

Por se tratar de um relato de experiência, não há informações que permitam a identificação de pessoas ou instituições, não havendo necessidade de submissão ao Comitê de Ética.

## RESULTADOS

A implementação da TLBP em Santos foi impulsionada pelos resultados promissores observados em sua aplicação inicial em um complexo hospitalar de pequeno porte. A partir dessa experiencia, foi realizado um diagnóstico situacional para identificar o número de pessoas com necessidade do uso de laser e o número de profissionais que poderiam ser capacitados. Com base nesse levantamento, foi realizada uma licitação no município de Santos para a contratação de um serviço de educação para a qualificação e habilitação de enfermeiras no uso do equipamento de laser, conforme o parecer da Câmara Técnica nº 04/2016/Ctas/Cofen^(12)^ e o parecer do COREN-SP nº 009/2018^(13)^ sobre a utilização do laser vermelho e infravermelho no tratamento de feridas realizada por enfermeiras.

A emenda parlamentar supracitada foi gerida pela Secretaria Municipal de Saúde e a capacitação foi realizada em uma universidade. O curso teve duração de 30 horas teórico-práticas e abordou o tratamento profilático e curativo de lesões tissulares agudas e crônicas com TLBP.

O conteúdo desenvolvido e ministrado nessa capacitação, abrangeu os seguintes temas: indicações e contraindicações do uso do laser; entendendo a luz; densidade de energia ou fluência; a luz e a interação com o tecido biológico; laserterapia fotodinâmica; laserterapia no tratamento da dor; laserterapia no crescimento ósseo; normas de biossegurança; ILIB (*Intravascular Laser Irradiation of Blood*); protocolos de cicatrização e dor; laserterapia *versus* coberturas; equipamentos disponíveis no Brasil; laserterapia em crianças; laserterapia nas lesões pós-parto; lesões orais e laserterapia; estética; laser cirúrgico; e estratégias para implementar a laserterapia e definir valores associados à prática. Essa abordagem garantiu que as participantes tivessem acesso a conhecimentos atualizados e a instrumentos de alta qualidade, para sua aplicação no campo profissional.

Trinta e três enfermeiras foram selecionadas para participar da capacitação, priorizando aquelas profissionais atuantes em unidades com maior demanda de curativos. Após a capacitação, essas profissionais foram alocadas em nove unidades de saúde, representando uma cobertura inicial de 28% do território municipal. Simultaneamente, uma central de curativos foi criada no ambulatório de dermatologia e estomaterapia, localizado no município de Santos, para o atendimento de casos mais complexos.

A expansão da TLBP para todas as unidades da atenção primária à saúde foi processada gradualmente, impulsionada pelos resultados positivos observados e pela articulação da equipe de enfermagem com os gestores municipais. Em 2019, dois anos após o primeiro processo formativo das enfermeiras, evidenciou-se a necessidade de expandir a TLBP para todas as unidades da atenção primária do município de Santos e, assim, foram apresentadas as experiências exitosas do projeto inicial para os gestores, como por exemplo, a diminuição e a redução do tempo das internações quando se utilizava o laser na cicatrização das feridas, além de proporcionar um atendimento mais ágil e capilarizado, respondendo de forma mais efetiva a alta demanda de usuários com lesões tissulares agudas ou crônicas.

No processo, foi elaborado o Projeto de Lei nº 233/2019^(14)^ circunscrito ao atendimento às pessoas com doenças vasculares e lesões decorrentes destas patologias, em todo o município de Santos. Como resultado, a Secretaria de Saúde de Santos ofereceu verba para a amplificação do projeto, possibilitando a compra de mais dois equipamentos, totalizando 11 unidades de saúde com equipamentos de laser, cobrindo 34% da área territorial do município. Nesse mesmo ano, ocorreu o primeiro Simpósio de Curativos na cidade.

A partir do sucesso da etapa inicial, foi realizada nova licitação em 2023. Foram realizadas, então, duas novas capacitações, oportunizando a participação de mais 82 enfermeiras, divididas em dois grupos: 41 no primeiro e 41 no segundo. No total, 115 enfermeiras (33 na primeira capacitação e 82 na segunda capacitação), que atuam na rede de atenção à saúde do município de Santos, foram habilitadas para o manejo da TLBP, fortalecendo a rede de cuidado e expandindo o impacto das atividades formativas.

O resultado foi a inserção das enfermeiras capacitadas em todas as unidades da atenção primária do município, possibilitando o atendimento de pessoas com feridas de difícil cicatrização, em salas especialmente dedicadas, utilizando a TLBP. Nessa direção, atualmente 100% do território é coberto com atendimento da TBLP, incluindo os dois hospitais e as três unidades ambulatoriais de especialidades.

Como desdobramento dessas ações, foi criada a Lei Municipal nº 4.483, de 14 de maio de 2024^(15)^, que instituiu o programa municipal de atendimento vascular. O objetivo é promover a ampliação das políticas públicas voltadas à prevenção e ao tratamento das doenças vasculares, proporcionando atendimento qualificado, especializado e humanizado às pessoas acometidas por doenças vasculares e lesões decorrentes por meio da central de curativos do município, que passou a contar com um cirurgião vascular e de enfermeiras especializadas da rede municipal.

Vale salientar, que todas as instituições municipais nas quais a TLBP é oferecida, estão interligadas e as enfermeiras recebem capacitação contínua por meio da educação permanente, para a padronização e uniformização das técnicas; de modo que os atendimentos sejam realizados conforme o aprendizado na capacitação oferecida. Nas capacitações, as abordagens estiveram centradas nos tipos de feridas mais frequentes nos serviços, que variavam de acordo com as especialidades: na especialidade vascular, predominam úlceras e feridas de pé diabético; na pediatria e saúde materno infantil, predominam os furúnculos.

Atualmente, está em construção, a criação de um protocolo, que será impresso como documento oficial da Secretaria da Saúde, para nortear as práticas e condutas profissionais no município.

## DISCUSSÃO

O município de Santos é pioneiro no Estado de São Paulo e no Brasil na implementação da tecnologia de terapia a laser em 100% das unidades da Atenção Primária à Saúde. Essa experiência, reorienta a atenção à saúde de acordo com as necessidades dos sujeitos (usuários/pacientes) ou grupos sociais e, para que ela possa operar, é necessária a comunicação com toda a rede de atenção à saúde, incluindo os seus administradores^(10)^. Isso só é possível, como evidencia o caso apresentado, devido à autonomia relativa concedida aos administradores municipais pela descentralização do poder oferecida pela política do SUS.

Nesse modelo de gestão democrática e coparticipativa, os gestores municipais atuam como agentes-chaves na implementação de políticas e incorporação de novas tecnologias nas práticas em saúde. Essas medidas organizativas de planejamento e de gestão, implicam a organização da rede municipal de serviços e respondem às necessidades de saúde individuais e coletivas dos sujeitos de seu município^(10,16)^. Essa autonomia é essencial para a distribuição de poder e corresponsabilidade entre gestores, profissionais de saúde e cidadãos na prestação de cuidados. Na experiência do município de Santos, o processo decisório foi compartilhado entre trabalhadores e gestores, orientando o cuidado de acordo com as necessidades de saúde e com a integralidade do cuidado^(10)^.

Nesse contexto, houve vinculação e comunicação entre os profissionais dos diferentes níveis de atenção, com a atenção primária, atuando como o principal ponto de atenção na rede. O objetivo da atenção primária é ser resolutiva e isenta de preconceitos (racial, sexual, religioso, de origem, entre outros), atendendo à população do território e desempenhando papel fundamental para a gestão da clínica^(17)^. Nessa direção, considera-se integrar e legitimar os diversos conhecimentos e práticas em saúde para o enfrentamento dos desafios e com a proposta de educação na saúde para os profissionais em seus processos de trabalho, por meio da educação permanente, considerando-se, ainda, a realidade onde estão inseridos^(18)^. Assim, cumpre-se o objetivo da rede de atenção à saúde: a atenção à saúde integral e humanizada dentro do SUS^(17)^.

A melhoria da atenção à saúde também é impulsionada pelas novas tecnologias, que promovem eficiência, acessibilidade e qualidade nos serviços em diversas linhas de cuidado. Nesse sentido, a TLBP representa a mais recente tecnologia implementada no município de Santos, voltada para o atendimento das doenças crônicas não transmissíveis, especialmente para pessoas com doenças vasculares e lesões decorrentes da diabetes^(15)^, por exemplo. Muitas doenças crônicas não transmissíveis não têm cura, o mesmo pode acontecer com feridas sem possibilidades de cura, neste caso, as pessoas portadoras destas feridas, também são chamadas de “pacientes crônicos”; todavia, independente do potencial curativo, essas pessoas podem e devem receber tratamento adequado e oportuno, a fim de manter sua autonomia^(1)^.

Essa experiência possibilitou a ampliação da carteira de serviços da atenção primária à saúde do município de Santos. Trata-se de um documento que norteia as ações de saúde na atenção primária brasileira, com forte reconhecimento da clínica. Portanto, a experiência apresenta a TLBP como uma tecnologia inovadora para o cuidado em saúde já inserida no SUS^(19)^. Além disso, esta terapia fortalece a capacidade resolutiva da atenção primária.

Essa inovação tecnológica e as ações das enfermeiras e da equipe evitam que os sujeitos circulem entre os serviços em busca de cuidado, engendrando uma fragmentação da atenção à saúde. Nesse processo, é importante ressaltar que a enfermeira e a equipe oferecem um cuidado partilhado e singular do sujeito. Caso o pessoa necessite de cuidados de maior complexidade, o plano de cuidado (tratamento) é compartilhado entre a pessoa cuidada, família, comunidade e gestores; isto, com diálogo acessível e oportuno, visando à qualificação da assistência^(10,20)^.

Destarte, este relato não contém dados numéricos sobre o atendimento, pois seu objetivo é a discussão do processo de implementação da nova tecnologia no SUS. O protagonismo das enfermeiras na implementação da TLBP, demonstra que a inovação tecnológica, aliada ao engajamento profissional e à gestão participativa, pode transformar a realidade da Atenção Primária à Saúde, promovendo um cuidado integral, resolutivo e centrado nas necessidades da população.

## CONCLUSÃO

A relevância deste relato é a iniciativa protagonizada pelas enfermeiras, que não apenas exemplifica a autonomia e a capacidade de inovação dos profissionais, mas também reflete a importância do envolvimento político e organizacional no aprimoramento dos serviços de saúde. A liderança das enfermeiras foi crucial para a introdução da TLBP, demonstrando que a descentralização do poder no SUS permite a implementação de soluções adaptadas às necessidades de saúde de acordo com o município. Essa abordagem proativa não apenas ampliou a carteira de serviços da atenção primária à saúde, mas também fortaleceu a integração entre os diferentes níveis de atenção, promovendo um cuidado integral e resolutivo.

O uso da TLBP como uma nova tecnologia no cuidado das feridas de difícil cicatrização, demonstra como as inovações tecnológicas podem ser incorporadas de forma eficaz na prática clínica diária, melhorando a qualidade de vida das pessoas e otimizando os recursos do sistema de saúde. A educação permanente das enfermeiras, assegura a continuidade e a qualidade do atendimento, diligenciando a garantia de que a nova tecnologia seja utilizada de maneira eficaz e segura.

A experiência de Santos poderá servir como um modelo de referência para outras regiões e ilustra o potencial das iniciativas locais em transformar a gestão da clínica no SUS. A colaboração entre gestores, profissionais de saúde e a comunidade é essencial para a criação de um sistema de saúde mais eficiente, acessível e humano.

# Translation: La laserterapia como tecnología asistencial en atención primaria

**DOI:** 10.1590/1980-220X-REEUSP-2024-0249es

**Authors:** Graziela de Paula Povrezan, Luzana Mackevicius Bernardes, Eduardo Carvalho de Souza, Rosimeire Angela de Queiroz Soares, Elda de Oliveira

**Affiliations:** 1 Universidade Católica de Santos, Programa de Pós-Graduação Stricto Sensu em Saúde Coletiva, Santos, SP, Brasil.; 2 Faculdade de Ciências Médicas da Santa Casa de São Paulo, São Paulo, SP, Brasil.

**Keywords:** Terapia por Láser, Atención Primaria de Salud, Atención de Enfermería, Organización y Administración

## Abstract

**Objetivo:**

Relatar la experiencia de implantación de la laserterapia de baja potencia en la Atención Primaria en Salud (APS) en el municipio de Santos.

**Método:**

Estudio descriptivo con marco temporal entre 2017 y 2024. Participaron enfermeros (especialistas y médicos generales) y gestores. El análisis se basó en el marco teórico de la gestión clínica.

**Resultados:**

La implementación de la terapia láser comenzó con una enfermera en un hospital pequeño. El éxito del proceso estuvo impulsado por la articulación política de la enfermera, el diálogo con los distintos niveles asistenciales y directivos, y la adquisición de equipos láser. Un total de 115 enfermeras fueron capacitadas para implementar la técnica en todas las unidades del SUS del municipio, con énfasis en la APS.

**Consideraciones finales:**

La experiencia demuestra el potencial de las iniciativas locales para transformar la gestión clínica en el SUS. La colaboración entre gestores, profesionales de salud y la comunidad es fundamental para un sistema de salud más eficiente, accesible y humano.

## INTRODUCCIÓN

El tratamiento de las heridas crónicas representa un reto para la salud pública mundial, que afecta a millones de personas y genera importantes costes para los sistemas sanitarios. Se calcula que la prevalencia de las heridas crónicas oscila entre el 0,18% y el 1,3% de la población, dependiendo del contexto y de los criterios diagnósticos utilizados^(1)^. Estas lesiones suelen estar asociadas a enfermedades crónicas como la diabetes y las enfermedades vasculares, e imponen molestias considerables a los sujetos, comprometiendo su calidad de vida y aumentando el riesgo de complicaciones graves como infecciones y amputaciones. Por ello, es prioritario en el ámbito sanitario establecer terapias eficaces que favorezcan la curación y minimicen el sufrimiento. Así, la Terapia Láser de Baja Potencia (TLBP) ha surgido como una alternativa tecnológica prometedora en este escenario, con el potencial de contribuir significativamente al manejo clínico de las heridas^(2)^.

La investigación en el uso y desarrollo de tecnologías sanitarias y la innovación se ha fomentado desde el año 2000, con la creación del Departamento de Ciencia y Tecnología, parte de la Secretaría de Ciencia, Tecnología e Insumos Estratégicos. En 2018, este departamento, junto con la Organización Panamericana de la Salud, lanzó la nueva agenda de prioridades de investigación del Ministerio de Salud^(3)^. El eje cuatro de la agenda aborda la importancia de realizar investigación en el ámbito del desarrollo tecnológico y la innovación en salud, especialmente en la Atención Primaria de Salud^(3)^, puerta de entrada preferente y coordinadora de la atención en el Sistema Único de Salud, con funciones clave para la integración de la Red Asistencial de Salud (RAS)^(4)^. Así, la TLBP, una de las innovaciones tecnológicas, se ha implantado en la atención a los usuarios de Atención Primaria^(5,6)^.

A nivel mundial, la TLBP se utiliza desde los años 80, cuando Tina Karu, importante biofísica de origen estonio, demostró los efectos de la luz de baja potencia sobre los componentes moleculares. Estos componentes moleculares, al absorber los haces de luz, muestran una activación a corto plazo del transporte de electrones, aumentando la síntesis de trifosfato de adenosina y reduciendo el pH intracelular. Estas reacciones fisiológicas constituyen la base conceptual de los efectos del TLBP, llevando a la célula a una condición de equilibrio^(7)^. La acción del TLBP favorece el proceso de cicatrización y la acción bactericida. Las ventajas en la práctica clínica incluyen el alivio del dolor, el control de procesos inflamatorios, el tratamiento de infecciones, la regeneración tisular y el drenaje de edemas, variando la intervención en función de las necesidades asistenciales de la persona. Además, el uso de TLBP reduce los costes hospitalarios en el tratamiento de lesiones cutáneas^(8)^.

En 2017, el primer uso de TLBP en el municipio de Santos fue implementado por una enfermera (estomaterapeuta y gestora hospitalaria) que comenzó a utilizar esta terapia, con su propio equipo, para tratar lesiones cutáneas en un complejo hospitalario del municipio. Las experiencias exitosas de esta enfermera han puesto de manifiesto la necesidad de ampliar esta intervención en la Atención Primaria de Salud. La implantación de la TLBP en los cuidados de la Red Asistencial está en consonancia con su propuesta de ofrecer servicios asistenciales que impliquen la promoción, prevención, diagnóstico, tratamiento y rehabilitación en salud colectiva e individual. El objetivo de este texto es, por lo tanto, relatar la experiencia de implementación de la TLBP en la Atención Primaria de Salud en el municipio de Santos.

## MÉTODO

Se trata de un estudio descriptivo del tipo relato de experiencia. Al socializar una experiencia, es necesario un abordaje crítico-reflexivo para suscitar el debate y posibilitar la reflexión, contribuyendo a la producción de conocimiento científico^(9)^. La descripción es temporal y transversal, abarcando el periodo de 2017 a 2024. El marco teórico del análisis se basa en la gestión clínica^(10)^, que destaca tres ejes estructuradores en los sistemas integrados de salud, como la red asistencial: el modelo asistencial; el modelo de gestión y la concepción de la educación sanitaria. Estos ejes proporcionan una base sólida para implantar prácticas sanitarias eficaces y coherentes y orientan los principios de la gestión clínica en una administración democrática y coparticipativa. La experiencia relatada se inició en un pequeño complejo hospitalario del municipio de Santos. Posteriormente, la iniciativa se difundió ampliamente por todo el municipio, situado en el litoral del estado de São Paulo, a 70 kilómetros de la capital. Santos tiene una población estimada de 433.656 habitantes y un índice de desarrollo humano de 0,840; cuenta con 32 unidades de atención primaria y en todas ellas trabajan enfermeras. La ciudad está dividida por el IBGE en ocho áreas o distritos sanitarios. En comparación con el resto del estado de São Paulo, los habitantes de la ciudad son mayores, es decir, tienen más de 60 años^(11)^, lo que corresponde al 22,6% de la población.El proceso de implantación de la laserterapia, como se mencionó anteriormente, fue coordinado por una enfermera que viabilizó el proceso burocrático e incluyó pasos fundamentales para garantizar su ejecución y calidad. La iniciativa contó con el apoyo de la administración sanitaria municipal y con un presupuesto obtenido a través de enmiendas parlamentarias destinadas a financiar el proyecto. Esta cuidadosa planificación fue esencial para garantizar que el contenido del programa se estructurase de acuerdo con los estándares de excelencia.Al tratarse de un informe de experiencia, no hay información que permita identificar a personas o instituciones, por lo que no es necesario someterlo al Comité de Ética.

## RESULTADOS

La implantación del TLBP en Santos fue impulsada por los prometedores resultados observados en su aplicación inicial en un pequeño complejo hospitalario. A partir de esta experiencia, se realizó un diagnóstico de situación para identificar el número de personas que necesitaban terapia láser y el número de profesionales que podrían ser formados. A partir de este relevamiento, se realizó una licitación en el municipio de Santos para contratar un servicio de educación para capacitar enfermeros en el uso de equipos láser, de acuerdo con el dictamen de la Cámara Técnica Nº 04/2016/Ctas/Cofen (Consejo Nacional de Enfermería)^(12)^ y el dictamen de COREN-SP (Consejo Regional de Enfermería de Sao Paulo) Nº 009/2018^(13)^ sobre el uso de láser rojo e infrarrojo en el tratamiento de heridas por enfermeros. La mencionada enmienda parlamentaria fue gestionada por la Dirección Municipal de Salud y la capacitación se realizó en una universidad. El curso tuvo una duración de 30 horas teórico-prácticas y abarcó el tratamiento profiláctico y curativo de lesiones tisulares agudas y crónicas con TLBP. El contenido desarrollado e impartido en este curso de formación abarcó los siguientes temas indicaciones y contraindicaciones para el uso del láser; comprensión de la luz; densidad de energía o fluencia; luz e interacción con el tejido biológico; terapia láser fotodinámica; terapia láser en el tratamiento del dolor; terapia láser en el crecimiento óseo; normas de bioseguridad; ILIB (*Irradiación Láser Intravascular de la Sangre*); protocolos de cicatrización y dolor; terapia láser *versus* coberturas; equipos disponibles en Brasil; terapia láser en niños; terapia láser en lesiones postparto; lesiones orales y terapia láser; estética; láser quirúrgico; y estrategias de implementación de la terapia láser y definición de valores asociados a la práctica. Este enfoque garantizó que los participantes tuvieran acceso a conocimientos actualizados e instrumentos de alta calidad para su aplicación en el ámbito profesional. Treinta y tres enfermeras fueron seleccionadas para participar en la formación, dando prioridad a las que trabajaban en unidades con mayor demanda de apósitos. Tras la formación, estos profesionales fueron asignados a nueve unidades sanitarias, lo que representa una cobertura inicial del 28% del territorio municipal. Al mismo tiempo, se creó un centro de curas en el ambulatorio de dermatología y estomaterapia, situado en el municipio de Santos, para tratar los casos más complejos. La expansión del TLBP a todas las unidades de atención primaria se llevó a cabo de forma gradual, impulsada por los resultados positivos observados y el enlace entre el equipo de enfermería y los gestores municipales. En 2019, dos años después del primer proceso de formación de las enfermeras, se hizo evidente la necesidad de ampliar el TLBP a todas las unidades de atención primaria del municipio de Santos, por lo que se presentaron a los gestores las experiencias de éxito del proyecto inicial, como la reducción y el acortamiento de los tiempos de hospitalización cuando se utilizaba el láser para curar heridas, así como la prestación de una atención más ágil y capilar, respondiendo con mayor eficacia a la gran demanda de usuarios con lesiones tisulares agudas o crónicas.

Como parte del proceso, se elaboró el Proyecto de Ley 233/2019^(14)^ para la atención de personas con enfermedades vasculares y lesiones derivadas de estas patologías en todo el municipio de Santos. Como resultado, el Departamento de Salud de Santos ofreció fondos para ampliar el proyecto, permitiendo la compra de dos equipos más, totalizando 11 unidades de salud con equipos láser, cubriendo 34% del área del municipio. Ese mismo año, se celebró en la ciudad el primer Simposio de Vendajes. Basándose en el éxito de la etapa inicial, se convocó una nueva licitación en 2023. Entonces se celebraron dos nuevos cursos de formación, que dieron la oportunidad de participar a 82 enfermeros más, divididos en dos grupos: 41 en el primero y 41 en el segundo. En total, 115 enfermeros (33 en la primera formación y 82 en la segunda), que trabajan en la red asistencial del municipio de Santos, fueron cualificados para gestionar el TLBP, reforzando la red asistencial y ampliando el impacto de las actividades de formación. El resultado fue la inclusión de enfermeros formados en todas las unidades de atención primaria del municipio, lo que permitió atender a las personas con heridas de difícil cicatrización en salas especialmente dedicadas utilizando TLBP. En la actualidad, el 100% del territorio está cubierto por la atención TLBP, incluidos los dos hospitales y las tres unidades ambulatorias de especialidades.

Como resultado de estas acciones, se creó la Ley Municipal nº 4.483, de 14 de mayo de 2024^(15)^, por la que se establece el programa municipal de atención vascular. El objetivo es promover la expansión de las políticas públicas destinadas a la prevención y tratamiento de las enfermedades vasculares, proporcionando atención calificada, especializada y humanizada a las personas afectadas por enfermedades vasculares y lesiones resultantes a través del centro de curas del municipio, que ahora cuenta con un cirujano vascular y enfermeras especializadas de la red municipal.

Cabe destacar que todas las instituciones municipales en las que se ofrece TLBP están interconectadas y los enfermeros reciben formación continua a través de la formación continuada para homogeneizar y estandarizar las técnicas, de forma que los cuidados se realicen de acuerdo con la formación impartida. En los cursos de formación, los abordajes se centraron en los tipos de heridas más frecuentes en los servicios, que variaban según la especialidad: en la especialidad vascular, predominan las úlceras y heridas por pie diabético; en pediatría y salud materno-infantil, predominan los forúnculos.

Actualmente se está elaborando un protocolo que será impreso como documento oficial por el Departamento de Salud para orientar las prácticas y el comportamiento de los profesionales en el municipio.

## DISCUSIÓN

El municipio de Santos es pionero en el estado de São Paulo y en Brasil en la implantación de la tecnología de laserterapia en el 100% de las unidades de Atención Primaria de Salud. Esta experiencia reorienta la atención a la salud de acuerdo con las necesidades de los individuos (usuarios/pacientes) o grupos sociales y, para que funcione, es necesaria la comunicación con toda la red de atención a la salud, incluidos sus administradores^(10)^. Esto sólo es posible, como muestra el caso presentado, debido a la relativa autonomía concedida a los administradores municipales por la descentralización del poder ofrecida por la política del SUS.

En este modelo de gestión democrática y co-participativa, los gestores municipales actúan como agentes en la implementación de políticas y en la incorporación de nuevas tecnologías en las prácticas de salud. Estas medidas de planificación y gestión organizativa implican la organización de la red municipal de servicios y la respuesta a las necesidades sanitarias individuales y colectivas de la población de su municipio^(10,16)^. Esta autonomía es esencial para la distribución del poder y la corresponsabilidad entre gestores, profesionales de la salud y ciudadanos en la prestación de los cuidados. En la experiencia del municipio de Santos, el proceso de toma de decisiones fue compartido entre trabajadores y gestores, orientando la atención de acuerdo con las necesidades de salud y la atención integral^(10)^.

En este contexto, hubo articulación y comunicación entre los profesionales de los diferentes niveles de atención, actuando la atención primaria como principal punto de atención de la red. El objetivo de la atención primaria es ser resolutiva y libre de prejuicios (raciales, sexuales, religiosos, de origen, entre otros), atendiendo a la población local y desempeñando un papel fundamental en la gestión clínica^(17)^. En esta dirección, se considera integrar y legitimar los diversos saberes y prácticas en salud para enfrentar los desafíos y con la propuesta de educación en salud para los profesionales en sus procesos de trabajo, a través de la educación permanente, considerando también la realidad donde están insertos^(18)^. De esta forma, se cumple el objetivo de la red asistencial: una asistencia sanitaria integral y humanizada dentro del SUS (Sistema Único de Salud de Brasil)^(17)^.

La mejora de la asistencia sanitaria también está impulsada por las nuevas tecnologías, que promueven la eficiencia, la accesibilidad y la calidad de los servicios en diversas líneas de atención. En este sentido, la TLBP representa la última tecnología implantada en el municipio de Santos, destinada a la atención de enfermedades crónicas no transmisibles, especialmente para personas con enfermedades vasculares y lesiones derivadas de la diabetes^(15)^, por ejemplo. Muchas enfermedades crónicas no transmisibles no se pueden curar, y lo mismo puede ocurrir con las heridas que no se pueden curar, en cuyo caso las personas con estas heridas también se denominan «pacientes crónicos»; sin embargo, independientemente de su potencial de curación, estas personas pueden y deben recibir un tratamiento adecuado y oportuno para mantener su autonomía^(1)^. Esta experiencia permitió ampliar la cartera de servicios de atención primaria de salud en el municipio de Santos. Se trata de un documento que orienta las acciones de salud en la atención primaria brasileña, con un fuerte reconocimiento de la clínica. Por lo tanto, la experiencia presenta a la TLBP como una tecnología innovadora de atención a la salud que ya ha sido incluida en el SUS^(19)^. Además, esta terapia refuerza la capacidad de la atención primaria para resolver problemas.

Esta innovación tecnológica y la actuación de las enfermeras y del equipo evitan que las personas se desplacen entre servicios en busca de atención, creando una fragmentación de la asistencia sanitaria. En este proceso, es importante subrayar que la enfermera y el equipo ofrecen una atención compartida y única para cada persona. Si la persona necesita cuidados más complejos, el plan de cuidados (tratamiento) se comparte entre la persona atendida, su familia, la comunidad y los gestores; esto se hace a través de un diálogo accesible y oportuno, con el objetivo de mejorar los cuidados^(10,20)^.

Por lo tanto, este informe no contiene datos numéricos sobre el cuidado, ya que su objetivo es discutir el proceso de implementación de la nueva tecnología en el SUS. El papel desempeñado por las enfermeras en la implementación de la TLBP demuestra que la innovación tecnológica, combinada con el compromiso profesional y la gestión participativa, puede transformar la realidad de la Atención Primaria de Salud, promoviendo una atención integral, resolutiva y centrada en las necesidades de la población.

## CONCLUSIÓN

Lo relevante de este informe es la iniciativa liderada por las enfermeras, que no sólo ejemplifica la autonomía y capacidad de innovación de los profesionales, sino que también refleja la importancia de la implicación política y organizativa en la mejora de los servicios sanitarios. El liderazgo de las enfermeras fue crucial para la introducción del TLBP, demostrando que la descentralización del poder en el SUS permite la aplicación de soluciones adaptadas a las necesidades sanitarias según el municipio. Este enfoque proactivo no sólo amplió la cartera de servicios de atención primaria, sino que también reforzó la integración entre los distintos niveles asistenciales, promoviendo una atención integral y resolutiva.

El uso de TLBP como nueva tecnología en el cuidado de heridas de difícil cicatrización demuestra cómo las innovaciones tecnológicas pueden incorporarse eficazmente a la práctica clínica diaria, mejorando la calidad de vida de las personas y optimizando los recursos del sistema sanitario. La formación continuada del personal de enfermería garantiza la continuidad y la calidad de los cuidados, asegurando que la nueva tecnología se utiliza de forma eficaz y segura.

La experiencia de Santos puede servir de modelo de referencia para otras regiones e ilustra el potencial de las iniciativas locales para transformar la gestión clínica en el SUS. La colaboración entre gestores, profesionales sanitarios y comunidad es esencial para crear un sistema de salud más eficiente, accesible y humano.
